# Shoulder ultrasound

**DOI:** 10.2349/biij.2.4.e58

**Published:** 2006-10-01

**Authors:** SP Tan, KJ Fairbairn, JE Kirk, WC Liong

**Affiliations:** 1 Radiology Department, National University Hospital Malaysia (HUKM), Kuala Lumpur, Malaysia; 2 Radiology Department, Queens Medical Centre Campus, Nottingham University Hospital, Nottingham, United Kingdom; 3 Radiology Department, City Hospital Campus, Nottingham University Hospital, Nottingham, United Kingdom

## INTRODUCTION

Shoulder ultrasound (US) can be done using various techniques. It is useful to establish a standard technique to be familiar with how normal structures appear. This will later assist in the recognition of pathology.

Shoulder US can be confusing to the beginner. Shoulder anatomy, as seen on real-time US, takes time to learn.

We demonstrate our standard technique for looking at rotator cuff pathology. Much of it was taught by our teachers or learnt from standard textbooks on musculoskeletal US.

We image the subscapularis (SUBSCAP), biceps (BT), supraspinatus (SST) and infraspinatus (IST) tendons. We look for impingement of the SST under the acromion process. We also image the acromioclavicular joint. We do not routinely image the teres minor tendon.

We hope to teach the beginner by using videos that show how each structure is scanned. US cine-loops show what is seen on the scanning monitor. Frozen US images with labelled line drawings are given to explain the structures seen.

This article is not comprehensive and includes no examples of pathology. We hope it will serve as a supplement to a standard textbook such as the one referenced [1].

## INSTRUMENT

We use a linear transducer – at least 7.5 MHz.

## TECHNIQUE

We routinely review radiographs of the shoulder of interest if available.

The following bony points are used to aid in positioning of the transducer (Movie 1):

- C – Lateral end of clavicle
- A – Acromion process
- Co – Coracoid process (not easily palpable but easily located by US as the bony projection inferior to the lateral third of the clavicle)

**Movie 1:** Bony landmarks used to aid in positioning of the transducer for shoulder US.

## SUBSCAPULARIS TENDON (SUBSCAP)

**Figure 1:**
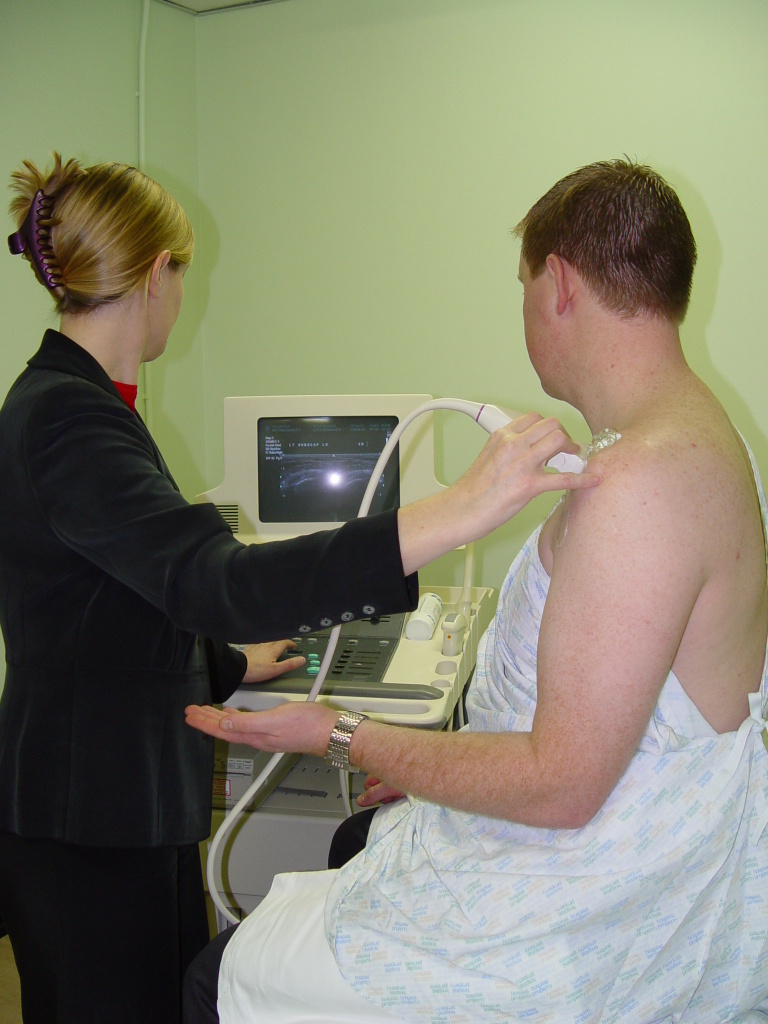
Starting position for imaging of the SUBSCAP. Patient sits facing the examiner. His arm is at his side, elbow flexed and forearm supinated.

- Patient sits on the examination couch facing the examiner. The patient’s arm is at his side and his elbow flexed. His forearm is supinated (Figure 1).
- The probe is placed axially at about the level of the coracoid process. You will see a longitudinal view of the SUBSCAP. The patient is asked to externally rotate his shoulder while keeping his arm by his side for a dynamic view of this muscle and tendon (Movie 2).

**Movie 2:** Longitudinal imaging of the SUBSCAP.

**Figure 2:**
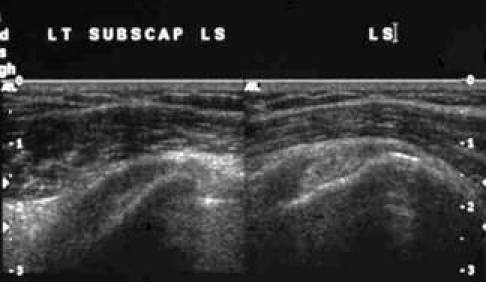
Frozen US image depicting a longitudinal view of the SUBSCAP in neutral position and in external rotation.

**Figure 3:**
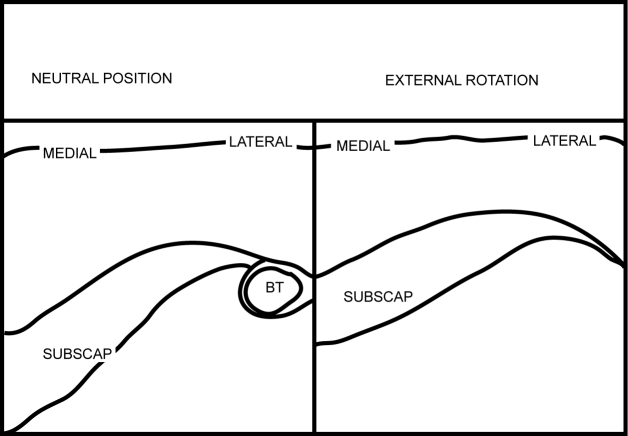
Labelled line drawing depicting a longitudinal view of the SUBSCAP in neutral position and in external rotation.

Cine-loop 1 shows the SUBSCAP in dynamic movement. Figures 2 and 3 are the frozen US image and the labelled line drawing respectively depicting a longitudinal view of the SUBSCAP in the neutral position and in external rotation.

**Cine-loop 1:** Longitudinal imaging of the SUBSCAP.

## BICEPS TENDON (BT)

- The patient is positioned as explained above for for SUBSCAP.
- The probe is placed axially at the anterior aspect of the shoulder at the level of the coracoid process slightly more lateral than for the SUBSCAP. You will see a transverse view of the BT in the bicipital groove. Pan up and down to follow the visible length of the BT. The probe is then positioned sagittally for a longitudinal view. Pan medial to lateral. You may need to tilt or ‘heel or toe’ the transducer to see the normal fibrillary pattern of the tendon (Movie 3).

**Movie 3:** Transverse and longitudinal imaging of the BT.

**Figure 4:**
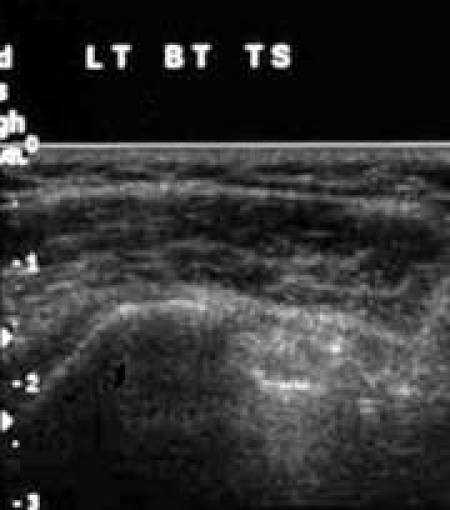
Frozen US image depicting a transverse view of the BT.

**Figure 5:**
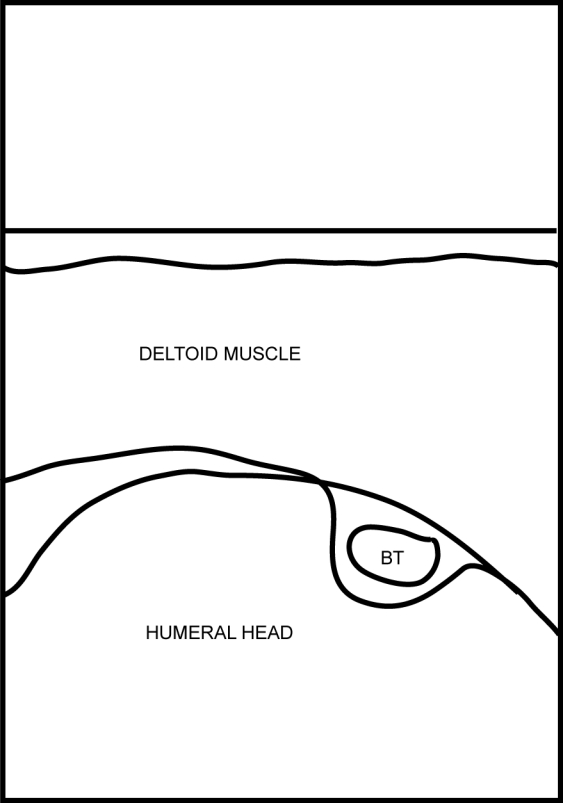
Labelled line drawing depicting a transverse view of the BT.

**Figure 6:**
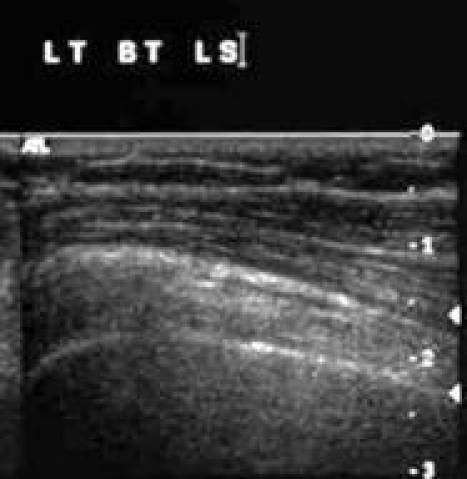
Frozen US image of a longitudinal view of the BT.

**Figure 7:**
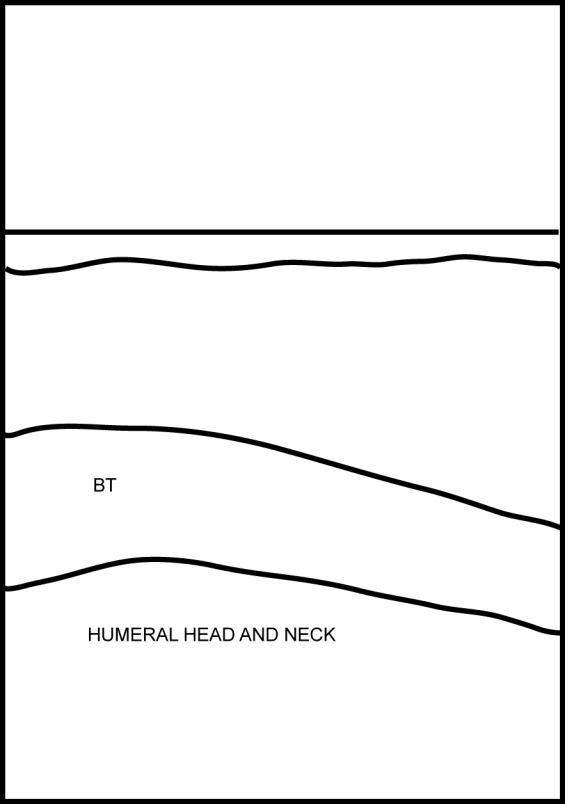
Labelled line diagram of a longitudinal view of the BT.

Cine-loop 2 shows the biceps tendon in transverse and in longitudinal views. Figures 4 and 5are the frozen US image and the labelled line drawing respectively, depicting a transverse view of the BT. Figures 6 and 7 are the frozen US image and the labelled line diagram of a longitudinal view of this tendon.

**Cine-loop 2:** Transverse and longitudinal imaging of the BT.

## SUPRASPINATUS (SST) AND INFRASPINATUS (IST) TENDONS.

These two tendons are positioned superior and posterior to the glenohumeral joint. They lie inferior to the acromion process. The SST is anterior and the IST is posterior. It is difficult to clearly separate these two tendons on US.

**Figure 8:**
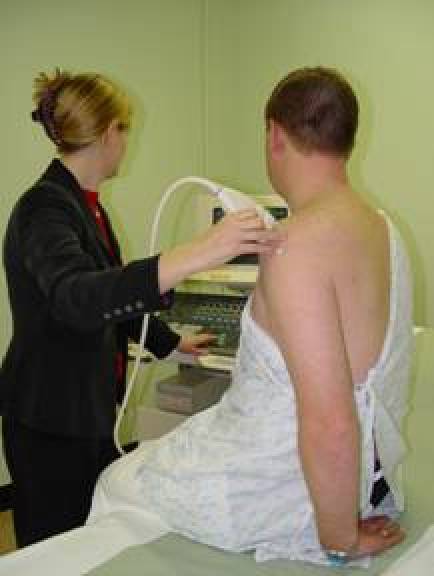
Starting position for imaging of the SST and IST. The patient rests the back of his hand on the couch behind him (waiter’s tip position).

- The patient is asked to rest the back of his hand on the couch behind him (waiter’s tip position) (Figure 8).
- Place probe in the sagittal oblique position just superior to the coracoid process. You will get a transverse view of the proximal SST with the anterior-most aspect of the SST marked by the BT. Pan posteriorly for the IST. Follow these tendons inferolaterally to see their insertion into the greater tuberosity of the humerus. Place probe in the coronal oblique position to get a longitudinal view of these tendons. Pan anteriorly for the SST and posteriorly for the IST (Movie 4).

**Movie 4:** Transverse and longitudinal imaging of the SST and IST.

**Figure 9:**
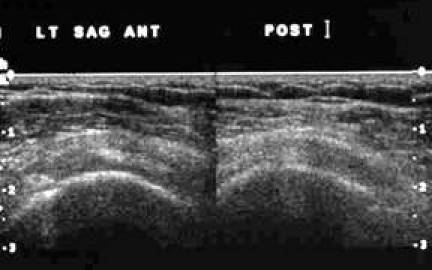
Frozen US image depicting a transverse view of the proximal SST and IST.

**Figure 10:**
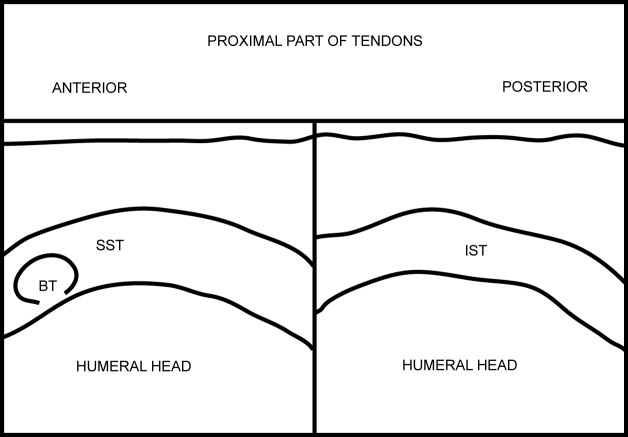
Labelled line diagram depicting a transverse view of the proximal SST and IST.

**Figure 11:**
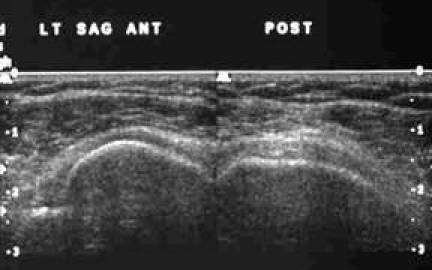
Frozen US image depicting a transverse view of the distal SST and IST.

**Figure 12:**
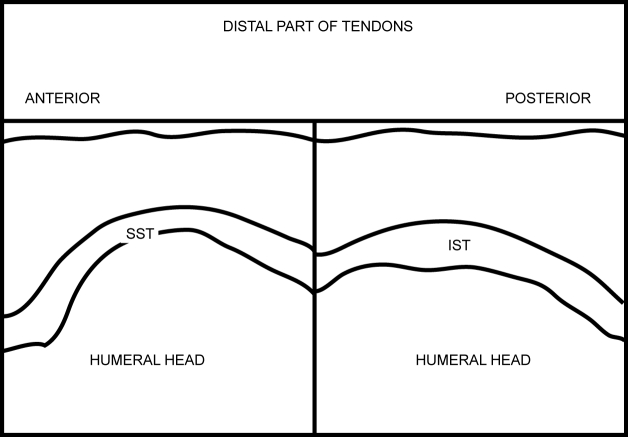
Labelled line drawing depicting a transverse view of the distal SST and IST.

**Figure 13:**
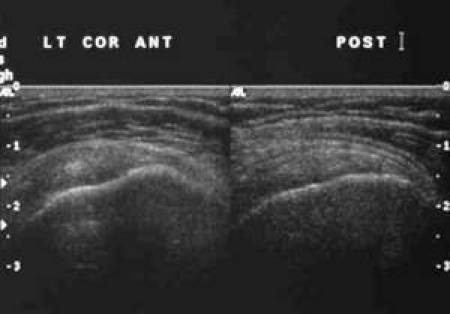
Frozen US image depicting a longitudinal view of the SST and IST.

**Figure 14:**
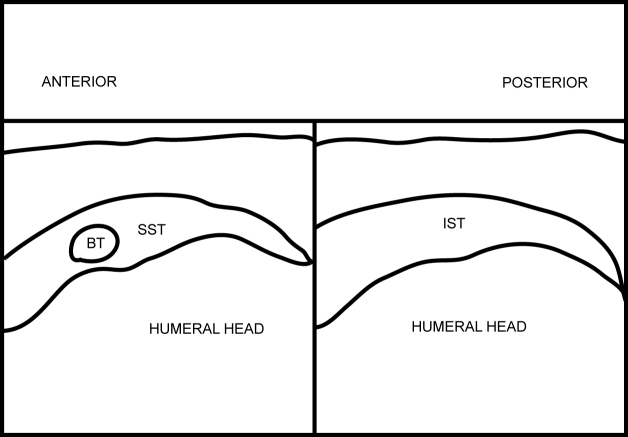
Labelled line drawing depicting a longitudinal view of the SST and IST.

Cine-loop 3 shows the SST and IST in transverse and longitudinal views. Figures 9 and 10are the frozen US image and the labelled line diagram respectively depicting a transverse view of the proximal SST and IST. Figures 11 and 12 are the frozen US image and the labelled line drawing respectively depicting a transverse view of the distal SST and IST. Figures 13 and 14are the frozen US image and the labelled line drawing respectively depicting a longitudinal view of these two tendons.

**Cine-loop 3:** Transverse and longitudinal imaging of the SST and IST.

## IMPINGEMENT

- Patient’s position as for examination of the SST and IST.
- Place probe in the coronal oblique position at the acromion process. You will see the SST in its longitudinal view lying just below the acromion process. Observe the smooth gliding of this tendon under the acromion process as the patient abducts his shoulder (Movie 5).

**Movie 5:** Manoeuvre to demonstrate impingement of the SST by the acromion process.

**Figure 15:**
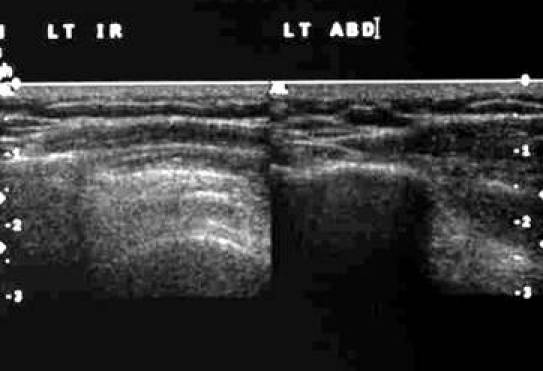
Frozen US images depicting a longitudinal view of the SST with the shoulder in internal rotation (IR) and adduction; and followed by shoulder abduction (ABD).

**Figure 16:**
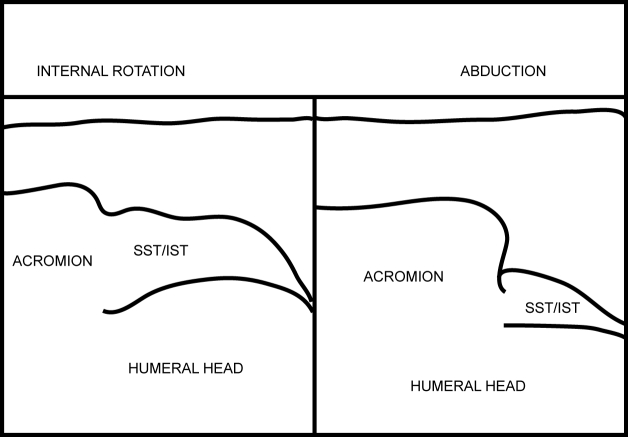
Labelled line drawing depicting a longitudinal view of the SST with the shoulder in internal rotation (IR) and adduction; and followed by shoulder abduction (ABD).

Cine-loop 4 shows the SST gliding under the acromion process. The starting position is with the shoulder in internal rotation (IR) and adduction followed by shoulder abduction (ABD). Figures 15 and 16 are the frozen US images and the labelled line drawing respectively depicting a longitudinal view of the SST with the shoulder in internal rotation (IR) and adduction; and followed by shoulder abduction (ABD).

**Cine-loop 4:** Longitudinal imaging of the SST to demonstrate impingement.

## ACROMIOCLAVICULAR JOINT (ACJ)

**Figure 17:**
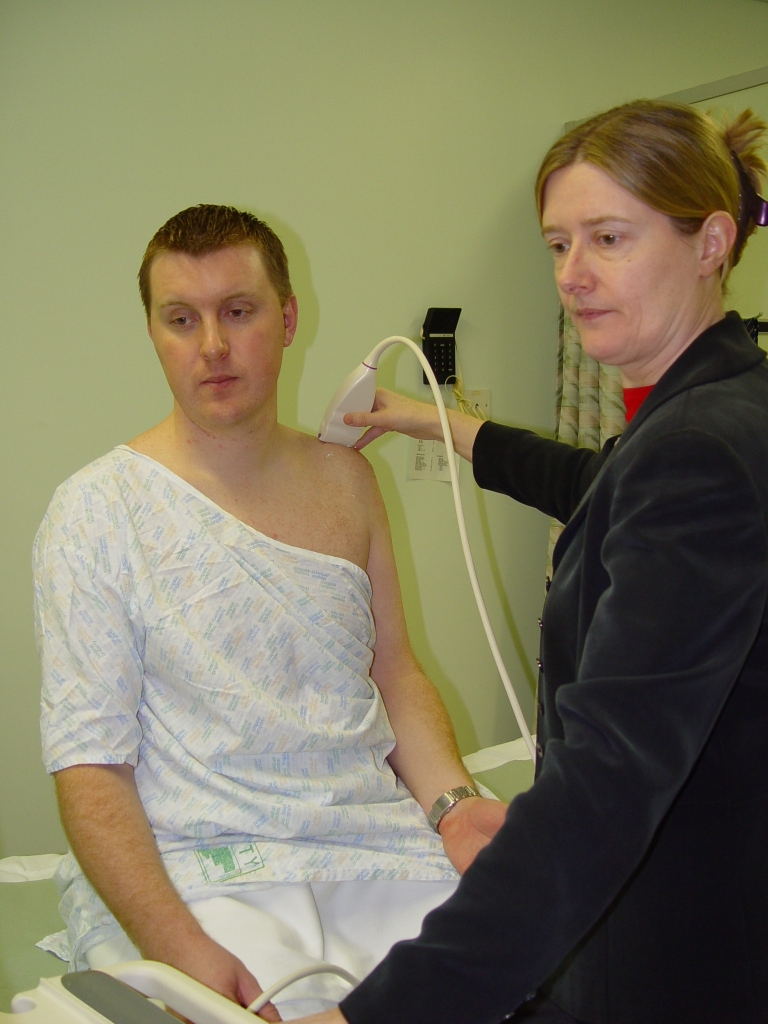
Patient and probe position for imaging the ACJ.

**Figure 18:**
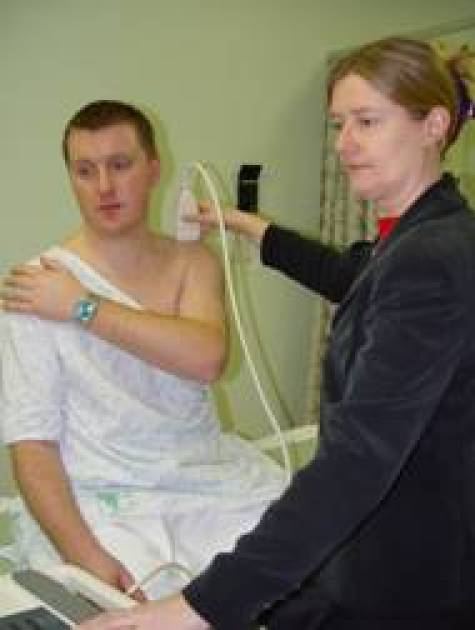
Scarf test. The patient moves his arm across his chest.

- Patient’s position as for examination of the SUBSCAP. Place probe in the coronal oblique position at the acromion process. Move probe slightly medially to visualise ACJ (Figure 17). Note width of gap.
- Ask the patient to move his arm across his chest so that his hand touches his opposite shoulder (Figure 18). Notice narrowing of the ACJ space (Scarf Test) (Movie 6). A positive test is when the patient feels pain at the ACJ with this manoeuvre.

**Movie 6:** ACJ Scarf test.

**Figure 19:**
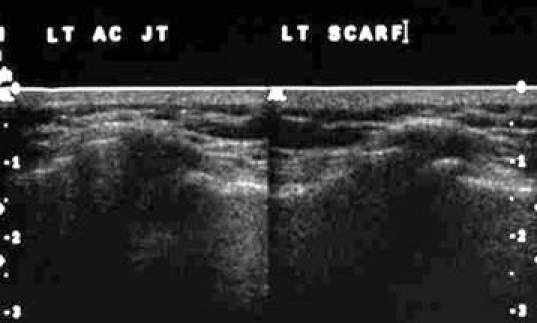
Frozen US image of the ACJ in the resting position and on doing the Scarf test.

**Figure 20:**
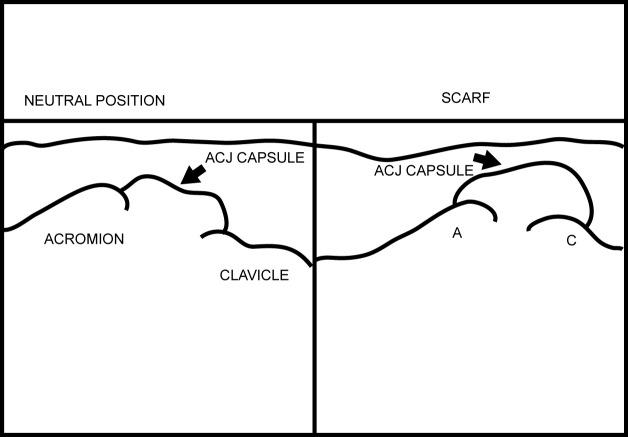
Labelled line drawing of the ACJ in the resting position and on doing the Scarf test.

Cine-loop 5 shows the ACJ as the patient does the Scarf test. Figures 19 and 20 show the frozen US image and the labelled line drawing respectively of the ACJ in the resting position and on doing the Scarf test.

**Cine-loop 5:** ACJ Scarf test. Note narrowing of the ACJ with adduction of the shoulder.
